# Stakeholders’ perspectives of comprehensive sexuality education in Iranian male adolescences

**DOI:** 10.1186/s12978-021-01084-0

**Published:** 2021-02-02

**Authors:** Keshvar Samadaee Gelehkolaee, Raziyeh Maasoumi, Seyed Ali Azin, Saharnaz Nedjat, Moslem Parto, Ilia Zamani Hajiabadi

**Affiliations:** 1 Department of Reproductive Health, School of Nursing and Midwifery, Tehran University of Medical Sciences, Tehran, Iran; 2 Nursing and Midwifery Care Research Center, School of Nursing and Midwifery, Tehran University of Medical Sciences, Tehran, Iran; 3 Reproductive Biotechnology Research Center, Avicenna Research Institute, ACECR, Tehran, Iran; 4 Department of Epidemiology and Biostatistics, School of public health, Tehran University of Medical Sciences, Tehran, Iran; 5 Faculty Member of Organization for Educational Research and Planning (OERP)-Research Institute for Education (RIE), Tehran, Iran; 6 Student Research Committee, Faculty of Medicine, Semnan University of Medical Sciences, Semnan, Iran

**Keywords:** Sexuality education, Adolescent, Stakeholders’ perspectives, Qualitative study, IMB model, Iran

## Abstract

**Background:**

A coherent sexuality education program for adolescents is part of their sexual and reproductive rights and can help them have a healthier future. Therefore, the aim of this study was to explore the perspectives and intervention preferences of Iranian stakeholders regarding comprehensive sexuality education (CSE) in male adolescents based on the IMB model (information, motivation, behavioral skills).

**Methods:**

This study was a qualitative study that was analyzed through a directed content analysis approach. Individual interviews and focused group discussions (FGDs) were used for data collection. The data were collected through in-depth semi-structured interviews with stakeholders in two schools and the Education Department in Sari and the Ministry of Health and Ministry of Education in Tehran from March 2019 to August 2019. Data saturation was achieved after 28 interviews and 1 FGDs with 9 participants. Finally, two sets of data were coded and analyzed using directed content analysis.

**Results:**

In this study, five themes emerged as (1) role of institutions; (2) role of organizations; (3) need for stakeholder’s partnership; (4) need for adolescent sexuality socialization management; and (5) need for enhancing the teachers’ professional competence, which seemed to influence the implementation of CSE in male adolescents. Participants also expressed a number of intervention preferences for CSE. The most important of these was the change in macro policies, helping to create a culture against all forms of violence and breaking the taboo of sexuality education for children and adolescents.

**Conclusions:**

The results of this study revealed the need for a CSE program for adolescents' sexuality socialization. The finding showed that teachers required training to enhance their professional competence about sexuality issues. Therefore, it is necessary to design and implement culture-appropriate skill based programs to enhance the teachers’ professional competence regarding the adolescents’ sexual health.

## Background

Many adolescents, especially those in developing countries, face challenges in terms of sexual and reproductive health and rights [1, 2]. Misconceptions regarding adolescent sexuality education, adults’ inattention to adolescents’ sexual health needs, and social and cultural barriers about sexuality education in some countries, including Iran, threaten their physical and mental health [3, 4]. Studies have shown that in order to achieve sexual health, which is part of the sexual and reproductive rights of individuals, governments should follow a gender-transformative approach that it new approach focuses on the participation of adolescent boys and men in reproductive health programs and gender equality [5, 6]. Studies investigating comprehensive sexual education have shown several benefits for adolescent reproductive health education, including delayed onset of sexual activity, increased use of contraceptive methods, reduced risky behaviors, reduced unwanted pregnancies, and reduced sexually transmitted infections (STIs) [7–9]. It seems that a set of factors such as being based on human rights, positive view of gender, having a learning-center educational approach, being based on culture and religion, and the involvement of all stakeholders in the program design are the reasons for its success [10, 11]. Stakeholders include adolescents, parents, educators, health policy makers, and religious leaders who play a key role in the adolescents’ sexual socialization to varying degrees [11].

It is a taboo to talk about sexual matters and romantic relations between a girl and a boy before marriage in conservative societies with a religious background such as Iran, hence their sexual health approach is emphasis on Abstinence-only-until-marriage [12]. The results of a cross-sectional study in Iran showed 27/7% male adolescents (ages 15–18) were reported sexual experience [13] while, culturally, having an intact hymen for an unmarried woman is a value [14]. This gender double standard, adolescents' lack of knowledge and skills about healthy sexual behavior and increased incidence of sexually transmitted diseases in adolescents indicates that the current approach about sexual education has failed and needs to be revised [8, 15–17].

One of the approaches with a positive attitude towards the psycho-sexual development of adolescents is comprehensive sexuality education (CSE), which challenges the attitude and requires various skills in addition to accurate and scientific knowledge [9]. According to UNFPA (2018), boys have less reproductive health knowledge than girls [18]. Moreover, studies have also shown that gender (boy) is a risk factor for high-risk sexual behaviors [19–21]. Access to knowledge and skills regarding the use of reproductive health services is the main need of Iranian male adolescents [22, 23]. The Government of the Islamic Republic of Iran was committed to controlling HIV as a means of promoting the health of the community. They have made several achievements in combating HIV/AIDS, with the focus on injectable drug users, but have countered a series of challenges and gaps in the determination of violence against women prevalence, gender equality, the increase of high risk behaviour in adolescents and sexual education for them [24]. Babayanzad et al. (2020) also underlined the need for sexual health education for students, teachers, instructors, and school authorities to meet the needs of the parents of adolescent boys in Iran [25]. Because of acquiring information and sexual health skills can help change adolescents' attitudes and responsible behaviors [26–28],so interventions based on information, motivation and behavioral skills model (IMB) are recommended for sexual health [28, 29].

This model was introduced by Fisher and Fisher 1993 as a general social psychological conceptualization to promote sexual health-related behaviors [30]. The construct of this model includes knowledge (specific behavior information and cognitive myths influencing decision-making), motivation (personal motivation that includes beliefs and attitudes toward a behavior, and social motivation that includes perceived social support or social norms) and behavioral skills (Objective skills and self-efficacy) [28]. The results of an international conference on Promoting Adolescent Sexual and Reproductive Health in Ecuador (2014) emphasized that research on adolescents should move towards context-based research [31]. In Iran, as a conservative Persian country, there is a scarcity in studies investigating contextual comprehensive sexuality education.

Therefore, the aim of the present qualitative study is to explore stakeholders’ perspectives of comprehensive sexuality education in Iranian male adolescences. The results of this study provide valuable ideas for designing and expanding CSE programs in schools.

## Methods

### Study design and participants

The present study was the second part of a study with a multistage mixed methods design that investigated the perspectives and intervention preferences of Iranian stakeholders regarding CSE in male adolescents based on the IMB model for the first time in Iran (The Islamic Republic of Iran is a Muslim-majority country in West Asia. It has a conservative cultural context such as Malaysia and Nigeria). This study was a qualitative study. Data was collected through individual interviews and focus group discussions (FGDs) and analyzed with directed content analysis approach. Participants included parents (3), teachers (9), instructors (4), and counselors (5) of Iranian male adolescents aged 11–19 years from different boys’ high schools in Sari (A city in northern Iran) and religious leaders (2) and policymakers of the Ministry of Education (6) and Ministry of Health in Tehran (Capital of Iran) (8), Iran. perspectives about adolescents’ sexual and reproductive health needs were also extracted in the first stage of the study using the scoping review (10).Twenty-eight subjects (22 men, 6 women) were interviewed and 9 (3 men, 6 women) participated together in FGDs. Purposive sampling with maximum variation in terms of age, gender, education level, occupation, position, expertise and work experience was used to ensure diversity in viewpoints as inclusion criteria. The purpose of the FGDs was to reach a consensus among stakeholders through interaction between opinions.

### Data collection

The interviews and FGDs were conduct based on interview guide and developed by the research team based on the theoretical framework of the IMB model from March 2019 to August 2019 [29]. All interviews were conducted by a Ph.D. candidate in sexual and reproductive health (first author) in a private room at the school or workplace. Each interview lasted about 60 min and the interviews were audio- recorded with the permission of the participants. The interviews continued until data saturation was achieved. The interviews began with general and open-ended questions followed by probing questions (The directed question for the main category) for clarification. After interviews with key informants, 1 FGDs was held with the presence of policy makers and experts (sexual and reproductive health specialist, social medicine and psycho-sexual fellowship, health psychologist and epidemiologist who were Faculty member of Ministry of Health and Ministry of Education and have executive position in government.

The FGDs were guided by two researchers in the Faculty of Nursing and Midwifery, Tehran University of Medical Sciences. One of the two researchers facilitated the discussions while the other recorded and took notes of verbal and nonverbal interactions. This meeting lasted about 135 min (Two 60-min sessions with a 15-min break). At the beginning of session, the study objectives and protocol, the results of the scope review, and the participants’ rights were explained to the subjects. They were also assured of data confidentiality. First, the participants expressed key points from their professional point of view. The debate continued on each issue until a consensus was reached. Topics agreed upon in this meeting: educational approach, educational content, type of intervention, how to evaluate the intervention.Finally, the final results were obtained by discussing and interacting with the opinions. An excerpt from the interview guide is provided below:

Open-ended questions were: What is in your mind when you are called for performing sexual education?

What is your idea about sexual health education for adolescents?

The directed question for the main categories of ‘information’ Motivation, Behavioral Skill:

What content should a sexual health education program include?

What makes you tend to teach sexual health program in school?

What infrastructure is needed to teach sexual health in school?

Other questions like “Can you explain with more examples?” were asked based on the answers for further clarification.

### Data analysis

The data were analyzed using the directed content analysis approach. The goal of this approach to content analysis is to develop a concept or to validate a theoretical framework [32]. Model or theory constructs can focus on the research question. The IMB model has been used in a variety of HIV risk-reduction interventions and has been shown to reduce sexual risk-taking behavior [26, 33]. Using model constructs, the study begins by identifying variables or key concepts as themes and sub-themes and cods were extracted from data [34]. In this model, the constructs can work independently or together to influence behavior change [26]. Therefore, the data were collected through interviews using targeted questions based on the IMB model constructs and continued with probing questions to explore the participants’ experiences and opinions about sexuality education programs. Audio-recorded interviews have been transcribed. All the transcripts were readied several times and their key points were highlighted. In the next step, the highlighted texts were encoded. Eventually, the codes were classified into themes and sub-themes based on the model constructs. The all process from data recording to transcription and coding was done Persian then translated to English.

### Trustworthiness

In this study trustworthiness were considered through credibility, transferability, conformability, dependability. An audit trail and audit process were used to achieve neutral or unbiased results [32]. The credibility of the data was established through spending enough time on data collection, member checking (the transcripts and codes were returned to participants to confirm any ambiguous codes), and triangulation methods for data collection (observation, interview, taking notes, and FGDs). Transferability was enhanced through thick description and maximum variation in sampling methods such as convenience, purposive, and snowball methods. In this study, double coding by two qualitative research methodologists who specialized in sexual and reproductive health was considered for conformability. They supervised the processes of coding and extraction of subthemes and themes also disagreement in coding was resolved by discussion and agreement in the presence of both. For dependability, external checking was conducted by four experts who were not members of the research team. In the following, the suggested interventions were categorized according to the IMB model to explore any perceived facilitator and barrier that influenced the implementation of adolescent sexuality skill based program in Iran [35]. All interviews were conducted in Persian. The codes and themes were extracted and then translated from Persian to English by the authors, and the expert translator approved the translated version.

This study received ethical approval from the Research Ethics Committee of Tehran University of Medical Sciences. In addition, authorities of the Department of Education in Sari agreed to cooperate in this study. After explaining the objectives, the method of study and the confidentiality of the information to the participants, they signed an informed consent and gave the permission to audio-record the interviews. Participants were assured that the interviews would be deleted after the data was extracted.

## Results

Twenty-eight subjects were interviewed and 9 subjects participated in the FGDs. The median age of the participants was 47 years (range: 34 to 60). The socio-demographic characteristics of the participants are presented in Table 1. The findings are supposedly based on five themes, namely (1) Role of institutions (2) Role of organizations (3) Need for stakeholders' partnerships (4) Need for sexuality socialization management (5) Need for enhancing teachers' professional competence which seemed to influence implementation of CSE in male adolescents. Finally, participants reportedly expressed a number of intervention preferences for CSE (Table 2).

**Table 1:** Demographic characteristics of participants (N = 36)

Characteristic	Participants
Age (med)	47
Gender (N. %)	
Male	25 (69)
Female	11 (31)
Education level (N. %)	
Bachelor	4 (11)
Master degree	10 (28)
P.H.D	18 (50)
Physician	4 (11)
Occupation (N. %)	
Teacher	12 (33)
Manager	5 (14)
Consultant	8 (22)
Politician	11 (31)
Work experience (N. %)	
10–14 years	4 (11)
15–19 years	5 (14)
20–24 years	10 (28)
> 24 years	17 (47)

**Table 2:** The main themes and sub-themes

Main themes	Sub-themes
The role of institutions	Family
	Educational institutions
	Cultural and religious institutions
The role of organizations	National Organization (Ministry of Education, Ministry of Health, Broadcasting, Welfare, Justice)
	International organizations
The need for stakeholder’s partnership	Reaching the common definition
	Applying changes in macro policies
	Designing and evaluating context-based educational content
The need for adolescent’s sexuality socialization management	Society's expectations and conflicts over sexual issues
	Adolescence vulnerability and the need to acquire skills
	Challenges and features of the symbol design
	Consequences of taboo sexual issues
The need for enhance professional competence of teachers	Appropriate educational content
	Choosing the suitable executive approach
	Skill-based teaching

In the IMB model, two constructs of information and motivation have a great influence on each other. In this study, their overlap was very high, so the themes extracted for these constructs were jointly reported. These themes included:

### Theme 1: role of Institutions (information and motivation)

All participants emphasized the importance of institutions because they believe that child rearing begins there. This theme was divided into three sub-themes include family, educational institutions, cultural and religious institutions, which reflected the participants' viewpoints on the factors related to the implementation of a sexuality education program in schools.

#### Family

The family is of particular importance as the first institution of child sexuality socialization. A male teacher who was the principal's advisor said:

“Education is formed primarily in the family and it is much better to be offered by the same-sex parent. Unfortunately, this form of education is not possible for boys in our society because the father does not have a very active presence in the home and school due to his economic responsibilities” (p.1, 55 y).

“…If the family disagrees with sex education at school, they oppose and resist. But if they are trained and aware, they will strengthen the educational efforts” (p.5, 42 y).

#### Educational institutions

Educational centers offer training in cognitive, emotional, and behavioral areas to adolescents, so they play an important role in their sexuality education. A school man manager said:

“We have to use all the capacities of our educational system to offer appropriate and correct education; we need to strengthen the role of teacher counseling” (p.7, 50 y).

" …Peer groups are very important, especially children who are somehow popular with other children, such as children who are elected for the school council. This child speaks and knows the rules and can influence other children” (p.9, 47 y).

#### Cultural and religious institutions

Cultural and religious institutions should be directly and indirectly effective in implementing the program through forming values and beliefs of the family and friends. In this way, they can be both barriers and facilitators in implementing sexuality education programs.

A clergyman who was adviser to the director of the Department of Education said: “Islam is a religion that emphasizes sexuality education. It is not right to use religion to intimidate and create guilt in order to instill personal ideologies, because of it drives adolescents away from religion and is also detrimental to everyone”(p.15, 43 y).

“…Unfortunately, culture sometimes causes physical and psychological harms to adolescents, for example, female genital mutilation, honor killings or puberty in boys in some African tribes”(p.27, 51 y).

### Theme 2: role of organizations (information and motivation)

At a macro level, organizations can play a significant role in implementing programs by pursuing sexuality education policies. They play their role by unifying the educational content and guaranteeing the implementation of the program. This theme was divided into two sub-themes include national organizations and international organizations.

#### National organizations

Each participant was named a number of organizations that they considered responsible for implementing the program. A woman who represented the adolescent education at the ministry of health said: “The first factor that hinders the implementation of a sexuality education program is the Ministry of Education, which has built a wall around itself and defines values for itself that are completely different from the needs of the society. It has also been weak in its interaction with the Ministry of Health” (p.24, 54 y).

Most of the participants considered the role of the Islamic Republic of Iran Broadcasting, as the only national medium in the country, very important in creating culture and breaking taboos. A male consultant mentioned**:** *“*The media, especially the radio and television, are also very important because their target group is the whole society. People become aware and do not resist sexuality education in schools” (p.9, 47 y).

“It is good idea to have sexuality education for all adolescents, including the sexual minority and the disabled. You should also have a program for disabled children supported by the Welfare Organization to reduce social harms by addressing their needs”(p.13, 57 y).

The majority of the participants believed that laws should be backed by legal sanctions and enacted based on human rights to protect their job security. A counselor who was also a therapist mentioned:“An adolescent has the right, as a human being, to be aware of his or her own sexual issues as well as other parts of his or her body” (p.6, 51 y).

“Laws should be revised according to the needs of the society. There is not much scientific work because policymakers believe that other sexual orientations are not natural and humane and are harmful to human survival. Moreover, from religious and legal points of view, people with other sexual orientations are guilty and deserve punishment and imprisonment, which means they practically do not consider any rights for the sexual minority group” (p.23, 47 y).

#### International organizations

Most of the participants believed that the experiences of successful countries and credible international organizations should be used because reproductive health needs of adolescents are the same all over the world, and the way each country meet them should be determined by according to its cultural context. The director of the research center of the Education Department said: “We don't need to do anything new; we are human beings like other people in the world, we have common needs; therefore, we need to use their experiences without prejudice and prepare our own educational content” (p.16, 43 y).

### Theme 3: need for stakeholder’s partnership (information and motivation)

Before designing any training program, a stakeholder analysis should be done to benefit from the existing opportunities to reduce threats. In this way, the opinions of all stakeholders will be taken into account and the possibility of implementing the program will be increased. In this regard, the comments of the participants were categorized in three sub-themes, including reaching the common definition, applying changes to macro policies, designing and evaluating context-based educational content.

#### Reaching the common definition

Depending on their specialization, each authority views the issue from a different perspective, claiming that they are doing the best they can for adolescents. Therefore, it seems that achieving a holistic view can meet the demands of all stakeholders. A psycho-sexology fellowship who was an effective member of the policymaking team said: “Fortunately there have been interactions between colleagues from the Supreme Council of the Cultural Revolution, the Ministry of Health, and the Vice Presidency for Women and Family Affairs in recent months. However, we have to see if the original text of the sexual health document is approved and if everyone can do what they are supposed to do in the document”(p.27, 50 y).

#### Applying changes to macro policies

In order to make changes at the macro level, the attitude of the society and policymakers toward the needs and dangers that threaten the target community should change. Accordingly, a male teacher that specialized in sociology said: “From politicians to teachers and parents, they all believe that men will not be harmed in a relationship, so there is no specific program for boys and men although they are more at risk” (p.14, 50 y).

#### Designing and evaluating context-based educational content

Education and educational content in any community should be written based on the values and beliefs of that community to be applicable. In this regard, the participants expressed: “An educational program should be age-appropriate, culture-based, and evidence-based, with proper evaluation during and the end of the program. Based on the results, it will be introduced in the school curriculum”(p.25, 53 y).

The director of the research center continued “ … A program should be piloted to correct the problems. The goals of the target group should be considered in the program, and then it should be implemented in more cities” (p.16, 43 y).

### Theme 4: need for adolescent sexuality socialization management (behavioral skills)

Sexuality socialization is a process through which individuals acquire cultural beliefs, values, symbols, meanings, and concepts related to sexuality. This theme consisted of four sub-themes, including society's expectations and conflicts regarding sexual issues, challenges and features of the SSCS, adolescent vulnerability and the need to acquire skills, challenges and features of the SSCS and consequences of sexual taboos.

### Society's expectations and conflicts regarding sexual issues

Increased information, attitude and educational gap between the generations have caused problems and lack of proper communication between parents, teachers and adolescents. This difference in expectations creates conflicts for the adolescents that should be managed and resolved. A policymaker said: “Old training methods are not suitable for training today's generation. In the communication age, kids have access to all the good and bad information at the touch of a button” (p.27, 50 y).

“Adolescents are confused by the conflict between formal education and the realities of the society. Also, the society's expectations from them are not proportional to their current situation”(p.6, 51 y).

### Adolescent vulnerability and the need to acquire skills

Adolescence can be the age of vulnerability due to the peak of hormones and lack of control over emotions. Therefore, training and acquiring skills can help adolescents to pass this phase with less damage and good experiences to continue their lives.

“The managers' propensity for sexuality education programs is unfortunately based on harm, while we should have created cultural sensitivity based on comprehensive management and a positive view of sexuality education 10 years ago” (p.27, 50 y).

"Our children often enter a relationship in an ambiguous environment based on their curiosity and personal experiences or what they learn from their peers without any skills, which is very unfortunate” (p.7, 50 y).

### Challenges and features of the SSCS (Student Social Care System)

In Iran, a new plan has been implementing to prevent social harms for three years. With the implementation of this plan, the existing problems and educational needs of teachers, parents and students have been partially revealed. Managing Director of Social Injuries expressed his experiences: “The symbolic plan is one of the good things that have started. In this plan, the managers' view of social harm has changed and their desire to cooperate has increased” (p.23, 47 y).

"The SSCS design does not have a separate item for sexual discussion but sexual education can be included along with other subjects. The families and teachers have asked to be trained in some areas because of the challenges they face in their lives.” (p.10, 52 y).

#### Consequences of sexual taboos

Taboos surrounding sexuality may have devastating effects on the community health. Concealment, embarrassment, and fear of stigma cause problems and lay the groundwork for further problems. A father in the position of boys’ school principal who had a 15-year-old son expressed:“Taboos make the statistics incorrect, because either the school master publishes wrong statistics out of fear or the students who have problems do not come forward.” (p.3, 40 y).

“Breaking taboos and talking about sexual issues can prevent many problems, which require the skills of managers and policymakers. They can help by creating a culture with education based on age, gender and culture.”(p.25, 53 y). In general, stakeholders are expected to modify incorrect sexual beliefs and attitudes in society through suitable education.

### Theme 5: need for enhancing the teachers’ professional competence (behavioral skills)

Professional competence is a complex of knowledge, attitude, and skills that enables a person to perform tasks successfully and solve problems. This theme had three sub-themes, including appropriate educational content, choosing a suitable executive approach and skill-based teaching.

#### Appropriate educational content

Appropriate educational content means educational content that is accepted by cultural norms, and is helpful in achieving predetermined goals (Like FLHE, SHARE and LLL curriculum). In this regard, a participant said: “We have to be very careful in preparing the educational content. It must be in accordance with our culture and age appropriate. Moreover, it should continue from the beginning of schooling until the last academic year.” (p.3, 40 y).

“… In preparing the content, clear concepts, photographs and examples of what is happening in real life should be used to make it attractive to students.” (p.3, 40 y).

#### Choosing a suitable executive approach

An executable method can help make the program more effective. Most of the available resources should be considered to determine the approach. The first one is for a trained specialist to teach sexuality education in schools, and the other is for several teachers to teach parts of the topic depending on their field of study. In this regard, a participant said: “Due to the interconnection of science, teachers from different fields should contribute to sexuality teaching.” (p.15, 43 y).

A health policymaker said: “In order for an expert to be able to teach an educational curriculum, there is a need for complete infrastructure in the country's education system, which is not currently available in our country” (p.27, 50 y).

#### Skill-based teaching

It is important to train professional teachers who can provide the right educational content correctly and skillfully. Skill-based teaching was one of the indicators of teachers' professionalism. In this case, one of the participants said so:

“We need to be able to provide a safe situation for students in the class to enter sexuality discussions without any fears”. (p.3, 40 y)

“A teacher must have mastered and applied life skills to be able to teach these lessons. They should also be able to use technology in teaching”. (p.10, 52 y).

“… Expertise, commitment, and effort are three important factors for people who want to teach in this field” (p.24, 54 y).

**Fig. 1:**
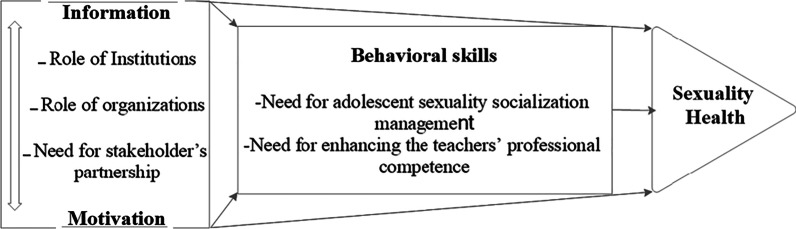
IMB model CSE program to achieve adolescent sexuality health

Figure 1 shows the model constructs and their effects on one another.

## Discussion

Our findings are supported by previous studies on the IMB health behavior change model for HIV prevention. Three themes, including the role of institutions, role of organizations, and need for stakeholder’s partnership, were identified in the information and motivation sections. Two themes were identified in the behavioral skills section, including the need for adolescent sexuality socialization management and need for enhancing the teachers’ professional competence. The participants also offered a number of suggestions for preferred interventions to improve the acceptance and implementation of CSE programs.

### Information

This section of the model consisted of two sub-categories, specific behavior information and cognitive myths influencing decision-making. Most of the participants believed that teachers, parents, and adolescents had very limited information about sexual health and that appropriate educational content needed to be designed to address the educational needs of each group separately. Since most of the resistance is due to misunderstanding the concept of sex education, it is better to use the term “sexuality education” and define it correctly. Because of issues related to sexual health are taboo according to cognitive myths, one of the suggested solutions is to break the taboo on sexual issues using educational capacities, religious leaders and the media. One of the cognitive myths held by all participants from parents to policymakers was that boys were less likely to be harmed in a relationship. For this reason, less attention has been paid to their reproductive health needs in educational programs. As reported previously [36–38], a combination of individual, family, religious, and socio-political factors can influence the people’s perceptions of sexuality education and its acceptance at the national level. Similar to previous studies, this study showed empowerment of parents regarding sexuality education is an effective intervention for promoting the adolescents’ sexual health [21, 25, 39, 40]. Therefore, sensitizing parents, encouraging their interaction with the school, and empowering them in order to reduce their resistance against formal education were among the suggestions proposed by teachers and policymakers. What may have been different in our study from other studies was the existence of educational conflicts between home and society. The reason seems to be that the family is influenced by the world of communication, while the educational policies of the society are based on religious views, regardless of the adolescents' access to the virtual world. In the present study as in previous studies [11, 41–45], concerns about job security and lack of life skills prompted the participants to suggest compulsory sexuality education in the formal education curriculum and extracurricular activities along with teacher empowerment. In line with the results of our study, Shipley et al. [46] reported talking about religious teachings and sexuality education is a challenging topic, especially in adolescents. The findings of the present study showed a dual role for religion. On the one hand, it has been suggested that strengthening the religious dimension in adolescents can help to achieve sexual health; therefore, this view can be used to expand the program similar to other studies in Iran. For example, the Najmabadi et al. non-use of religious potential was cited as a challenge for adolescents accessing health services [12]. Also, a qualitative study conducted by Bahrami et al. mentioned having strong religious convictions as a factor that promote and impede other-sex friendships in Iranian adolescent girls [47]**.** Somefun et al. studied on religiosity and sexual abstinence among Nigerian youths. The results of their study showed youth who were highly religious had significantly higher odds of abstaining compared to their counterparts who were not religious [48].

On the other hand, it has a deterrent role because from the viewpoint of conservative religious policymakers, the only acceptable approach to sexuality education is abstinence until marriage, as they believe that talking about safer sex or contraception methods may persuade adolescents to engage in sexual activity [3]. Therefore, religious beliefs have been regarded as a barrier in implementing such programs in some studies [3, 49]. Our study revealed similar to the findings of some studies that cultural and religious ceremonies can play a dual role in sexuality socialization. They can be pleasant experiences, such as the celebration of puberty and entering adulthood. Moreover, they can be harmful to sexual and reproductive health, such as female circumcision, honor killings or harming boys to prove masculinity by doing violent work [40, 50, 51]. Given the sub-theme of macro-policy change, policymakers should strive to adopt policies to create a culture in favor of reducing violence in society. The participates discussed the roles of the Ministry of Education, Ministry of Health, and Welfare Organization in establishing common research priorities, implementing research achievements, registering and presenting accurate statistics and information, and providing reproductive health services to adolescents with special needs such as the disabled people and sexual minorities, which were consistent with previous studies [11, 52] also Taghizadeh et al. showed a lack of coordination between the reproductive health needs of people with disabilities and the provision of services to them due to a lack of social and governmental support for the challenges of women with disabilities [53]. Revising the laws according to the needs of the society, prevention of school victimization and informing the judges about the differences in the issuance of sentences for different sexual orientations (homosexual, gender dysphonia), sexual offenders and sexual patients (paraphilia) were also suggested as the reasons for cooperating with the judicial organization [11, 40, 54]. Our findings, in accordance with previous studies [25, 42, 55], indicated a dual role for the media, social network and cyberspace. Similar to what Asrese et al. said, homogeneous networks were more likely to have high-risk sexual behaviors of adolescents, but engaging in sexuality discussion networks protected high-risk sexual behavior [55]. The participants suggested that the media, especially the state television, should take measures to break the taboo around sexuality. The adolescents' easy access to sexually explicit and pornographic media, on the other hand, can promote violence and high-risk behaviors. In agreement with the goals of international organizations, the findings of this study also showed that these organizations can be used to design and expand comprehensive sexuality education programs [6, 10, 18].

### Motivation

This section also consisted of two sub-categories, including personal motivation (motivation for a particular behavior, confidence in the outcome of the intervention) and social motivation (perceived social support, social norm for engaging in a behavior). The results of the following studies are in line with the results of the present study. For personal motivation, these include: strengthening religious beliefs [48], using peer groups [56], breaking taboos [57], preparing educational content [58], and acquiring life skills can motivate parents and teachers to engage in sexual health issues; moreover, they can motivate adolescents to engage in healthy behaviors [11]. For social motivation, these include: the participants proposed establishing youth-friendly centers [59, 60], moderating the society's expectations from adolescents, holding celebrations for entering adulthood, setting up virtual education networks, enacting right-based laws, and giving teachers and administrators access to accurate statistics and information [61].

Similar to previous studies conducted in countries with a similar cultural and religious background [42, 62–65], many participants considered the stakeholders reaching a common definition as one of the most important factors in implementing such programs. It is necessary to adopt a holistic and positive view of sexual issues with a multidisciplinary approach to policy-making, designing, implementing, and expanding sexuality educational programs.

### Behavioral skills

This section also consisted of two sub-categories, including strengthening the individual's objective skills and increasing perceived self-efficacy. In this study, two skills were identified by participants. One of the skills needed for policymakers was the ability to make sound decisions about sexuality education for adolescents. Due to the process of sexuality socialization affects all aspects of people's lives; policymakers always pay attention to sexuality education [62–64]. Therefore, adopting an appropriate approach in the field of adolescent sexuality education has been considered as a political power for policymakers. Due to the generation gap and the rapid cultural change, the society's expectations from adolescents do not meet their needs, so there are problems and conflicts between adolescents and adults regarding sexual matters that need to be managed,which was also discussed in some previous studies [63, 66]. This study, like previous studies, found that the taboo around sexual matters prevented teachers and parents from talking about them [62, 63]. This makes adolescents obtain their information on sexual matters from unreliable sources, which increases their vulnerability [6, 67]. The SSCS plan is now being implemented in Iran to prevent social harms to students. However, like other plans [45, 68], it faced challenges in the beginning. The subjects who participated in the pilot project discussed the benefits of the SSCS and hoped for its continuation. Therefore, it seems that applying the results of this study can help identify the weaknesses of the program and eliminate it.

Other skills are needed for teachers to be committed to the program and to teach it properly. In this regard, numerous studies have found that enhancing the teachers' professional competence is a key factor in sexuality education programs [69, 70]. An appropriate educational content can encourage the teacher to acquire the skills needed for teaching [6, 11]. Therefore, similar to the previous studies, the participants in the present study also emphasized this important point. Regarding the importance of choosing an executive approach to sexuality education, most of the participants stated that all teachers should acquire the necessary skills in this area and that the role of teacher counseling should be strengthened. As previous studies have suggested, CSE should be delivered by well-trained teachers who are willing to teach [11, 25]. A combination of knowledge, attitude, and skills-based learning is critical for empowering young people and enabling learners to take action [5, 11, 71].

In general, the qualitative design of this study provided an opportunity to discover the views and suggestions of Iranian stakeholders regarding the implementation of a comprehensive sexuality education program. In the following, we intend to design a culture and skill-based educational intervention for teacher empowerment based on the IMB model.

### Limitations

This study had the potential limitations of qualitative studies. The first limitation regarding the selection of participants was due to the lack of access to some managers and policymakers. In this regard, we tried to set the time and place with them from the prior time. We tried to reduce selection biases by conducting interviews among the diversity group. Second, it was difficult for them to talk about sexual issues because it is taboo. We also solved this problem by making sure the information was kept confidential. Despite the above limitations, interviewing key informants in ministries and designing the study based on a theoretical framework helped us to obtain an overview of the situation. Because of the data in this study highlighted the types of conflict in adolescence, it can be suggested to add conflict resolution skills as a topic to the key concept of International Technical guidance on sexuality education (No. 5). The findings of this study will be useful to policymakers in macro planning as well as educators, parents, and adolescents.

## Conclusion

The results of this study revealed that a correct perspectives and definition of comprehensive sexuality education is one of the key points that should be achieved through the stakeholder’s partnership. Disagreements between health professionals and policymakers over sexual issues due to taboos have affected the efforts to design and implement such programs. Although each policymaker confirmed the need for sexuality education, their collective opinion about major decisions shows the opposite in practice. These results indicate that culturally appropriate sustainable comprehensive model of sexual education and service for adolescent still is a challenge. Therefore, putting more effort into adolescent sexuality socialization can be a step towards achieving their sexual and reproductive health and rights.

### Implications

The results of this study provide the evidence to design and implement culture-based educational programs to address policymakers and educators regarding sexuality health in male adolescents.

## Data Availability

The data set are accessible by the corresponding author on request.

## Abbreviations

CSE: Comprehensive sexuality education
IMB: Information motivation behavioral skills
SSCS: Student social care system
FGDs: Focused group discussions
HIV: The human immunodeficiency viruses
AIDS: Acquired immunodeficiency syndrome
FLHE: Family life and HIV/AIDS education curriculum
SHARE: Sexuality, health, and responsibility education curriculum
LLL: Long live love curriculum
